# Genomic Variability Survey in *Ilex aquifolium* L., with Reference to Four Insular Populations from Eastern Europe

**DOI:** 10.3390/ijms252413593

**Published:** 2024-12-19

**Authors:** Ciprian Valentin Mihali, Alexandru Eugeniu Mizeranschi, Daniela Elena Ilie, Ludovic-Toma Cziszter, Radu Ionel Neamț, Andreea Ștefania Anton, Endre Mathe, Bence Pecsenye, Viviane Beatrice Bota, Violeta Turcuș

**Affiliations:** 1 Research and Development Station for Bovine Arad, 310059 Arad, Romania; alexandru.mizeranschi@scdcbarad.ro (A.E.M.); daniela.ilie@scdcbarad.ro (D.E.I.); radu.neamt@scdcbarad.ro (R.I.N.); andreea.anton@scdcbarad.ro (A.Ș.A.); 2 Faculty of Medicine, “Vasile Goldiș” Western University from Arad, 310025 Arad, Romania; mathe.endre@uvvg.ro (E.M.); bota.viviene@uvvg.ro (V.B.B.); turcus.violeta@uvvg.ro (V.T.); 3 Institute for Advanced Environmental Research, West University of Timisoara, 300086 Timisoara, Romania; 4 Faculty of Bioengineering of Animal Resources, University of Life Sciences “King Mihai I” from Timisoara, 300645 Timișoara, Romania; ludoviccziszter@usvt.ro; 5 Faculty of Agricultural and Food Sciences and Environmental Management, University of Debrecen, 4032 Debrecen, Hungary; 6 Institute of Nutrition Science, Faculty of Agricultural and Food Sciences and Environmental Management, University of Debrecen, 4032 Debrecen, Hungary; pecsenye.bence@agr.unideb.hu; 7 Faculty of Biology, “Alexandru Ioan Cuza” University of Iași, 700505 Iași, Romania; 8 Centre for Mountain Economy (CE-MONT), National Institute for Economic Research “Costin C. Kirițescu”, Romanian Academy, 725700 Suceava, Romania

**Keywords:** *Ilex aquifolium* L., genome analysis, short reads, Eastern Europe, Romanian insular population

## Abstract

Cosmopolitan in the western areas of Europe as well as on other continents, the Ilex genus is interesting for its genetic, phenotypic, and biogeographic variabilities. Its insular/local distribution, according to existing data on the periphery of the central and southern European areas, represents a suitable case study with reference to the adaptive plasticity or acclimatization of the *Ilex aquifolium* L. species to new climatic conditions. The aim of the present study was to analyze the genetic variability at the genome level in four insular populations of *Ilex aquifolium* L., i.e., in three spontaneous populations from Romania (RO), Serbia (SR), and Bulgaria (BG) and a cultivated population from Hungary (HU). According to the obtained results, the most genetically similar populations among the four considered in this study were those from SR and RO. Genetic variation overlapped genes that were generally associated with metabolic regulation/transport factors, water, and abiotic stress factors. The analysis of single-nucleotide polymorphisms (SNPs) at the levels of the chloroplast and mitochondrion, from the point of view of their distributions at the gene level, identified two clusters: one that includes the native populations (BG, SR, and RO) and a second one including the cultured population from HU.

## 1. Introduction

*Ilex aquifolium* L. is a dioecious, woody, evergreen species shrub or tree with an average height of up to 10 m, belonging to the Aquifoliaceae family [1]. More than 660 subspecies are described within the genus Ilex [2]. Currently, the Aquifoliaceae family is placed in the order Aquifoliales. Initially, the classification of the genus was achieved through studies of floral unit–cyme analysis (years 1949/50) [3,4] and, later, by molecular phylogeny studies (in the 2000s) [5].

The species/subspecies of the genus Ilex have diverse uses, being sources for the production of some tea drinks consumed in South America (yerba mate—*I. paraguariensis* **A**.**St.-Hil**.—and guayusa—*I. guayusa Loes*., with caffeine contents), China (’kudingcha’—bitter spike tea—*I. latifolia* **Thunb**., and *I. kaushue* **S.Y. Hu**, without caffeine or other methylxanthines [6]), and the United States (yaupon—*I. vomitoria* **Sol.** ex **Aiton,** with caffeine, and *I. opaca* **Aiton**—Appalachian tea, without caffeine) [7]. They are sources in the production of medicines (active principles used in traditional Chinese medicine) with protective effects on the cardiocirculatory system, as well as anti-tumor, antioxidant, anti-inflammatory, lipid metabolism regulatory, anti-microbial, and anti-diabetic effects [8]; similar studies are also presented in the cases of South American species [9]. *Ilex aquifolium* L. has an ornamental importance in urban landscaping and is socio-culturally associated with the celebration of some holidays [10].

The species of the Ilex genus spread from the tropics to the temperate zones, the area covering the American continents (the native residence of the genus Ilex is in South America, and the native or invasive residence—invasive in the Pacific Northwest wildlands—is in North America, *Ilex aquifolium* L.) [11], North Africa (Tunisia and Morocco), Central East Asia, Australia, and New Zealand [12]. It is present in almost all of Europe, originating in the Mediterranean basin, as in the Ilex–Taxus complex [13]. This species is mentioned in the 2013 IUCN Red List of Threatened Species, listed as a least-concern species. In agreement with the IUCN, on the European continent, *Ilex aquifolium* L. is present as a native resident species in the Scandinavian countries, the British Isles, from Western Europe descending to the South in Spain, and on the Balkan peninsula, in Serbia, Bulgaria, and Greece. The same list mentions that in Hungary, *Ilex aquifolium* L. exists as a cultivated species, while in Romania, it has an uncertain status regarding the native residence [14]. At the same time, other scientific sources mention the native uncertain residence of the Romanian insular population or the likelihood that it is a native resident species in Romania [15].

The first records about the *Ilex aquifolium* L. presence in the territory of Romania, near the village of Zimbru, were presented in 1893 as a part of an inventory study of forest species, where *Ilex aquifolium* L. was mentioned together with other woody species, such as *Acer platanoides* L., *A. pseudoplatanus* L., *Fagus sylvatica* L., and *A. tegmentosum* **Maxim** [16,17]. During the 90’s *Ilex aquifolium* L. was presented in Romania as a protected species [18]. As a consequence, in 1999, it was preserved within the Dosul Laurului Nature Reserve (0.33 km^2^), with GPS coordinates 46°23055.500 N, 22°22050.900 E, located in the Codru-Moma Mountains, Zimbru population, Gurahonț Commune, Arad County [19].

There are studies of molecular analysis and the phylogeny of the Ilex-genus species at the levels of the chloroplast (matK, rbcL, atpB-rbcL, trnL-F, psbA-trnH, and rpl32trnL) and nuclear sequences (ITS and nepGS) [20]. Genomic analysis and de novo reconstruction, with annotations of the chloroplast genome based on seven different species belonging to the Ilex genus from China (not including *Ilex aquifolium*) [21], as well as phylogenetic analysis between species from the Hong Kong region were carried out [22]. The analysis/construction of a complete chloroplast genome and phylogenetic analysis studies were carried out on an Ilex ‘Beryl’ hybrid between *Ilex cornuta* and *Ilex latifolia* [23] and on Ilex ‘Tall Boy’, a hybrid between *Ilex aquifolium* and *Ilex latifolia*, respectively [24]. In DNA methylation studies (on *Ilex aquifolium* L.) based on methylation-sensitive amplified polymorphisms at the level of the entire genome, a decreasing methylation tendency was observed between leaves without thorns and those with thorns, with a correlation between the DNA methylation mechanism and phenotypic plasticity [25].

A popular option for genomic DNA analysis is represented by SNPs (single-nucleotide polymorphisms). Genomic analyses by intraspecific SNP markers were performed in different studies with the aim of discriminating between different cultivars in *Lactuca sativa* L. [26], in biodiversity phylogeny studies between different yeast strains [27], or in association studies between genetic structure and productive morphological characteristics in species of economic interest [28]. The literature also mentions studies of the phylogeny and distribution of SNPs correlated with their effects at the gene level in cultivated trees (Prunus sp.) [29] or in economic species with importance in perennial forest tree resistance to various diseases [30] by NGS (next-generation sequencing). Currently, no research studies have been performed in which SNPs were used (through NGS) as a tool in population genetic analysis on the species *Ilex aquifolium* L.

Studies that “started” from leaf material using long reads in NGS technology (PacBio CLR and HiC sequencing) produced the de novo construction of new genomes (in the high quality–deep coverage range from 40× to 230×) in plants belonging to Eucalyptus and Arachis species [31]; in addition, using WGS (whole-genome sequencing), evolutionary history analyses in endangered plant species (*Acer catalpifolium* **Rehd.**) [32] were performed.

WGS analyses by short reads are used to obtain fragments with a high reading accuracy with the aim of constructing/reconstructing DNA/RNA sequences or cellular organelle genomes (chloroplasts and mitochondria) [33]; using WGS technology, in combinatory reading mode (long reads combined with short DNA reads), the outputs are analyzed in phylogeny/biodiversity studies in different species [34], plant breeding [35], or diagnosis [36]. WGS projects by NGS (short/long reads) in the Ilex genus were mostly carried out in species from Asia/South America, areas corresponding to speciation/diversification points [37]. Present species in genomic analysis by NGS are *Ilex asprella* (online data at the chromosomal assembly level) [38], *Ilex chinensis* **Sims.** (at the scaffold assembly level, without online publication of the assembly but which can be obtained upon request from the authors) [39], *Ilex polyneura* L. (online data for assembled chromosomes with annotations) [37], and *Ilex paraguariensis* **A.St.-Hil.** (online data at the scaffold assembly level) [40].

Currently, there are no present studies with reference to entire-genome sequencing by NGS (long or short reads) in the *Ilex aquifolium* L. species. The present study had several aims, the first of which was the entire-genome sequencing of the species *Ilex aquifolium* L. through NGS by short reads, the most representative and cosmopolitan species of the genus present in Europe. The WGS was performed on four DNA samples belonging to four different island locations, Romania (RO), Hungary (HU), Serbia (SR), and Bulgaria (BG). The second purpose of the WGS of *Ilex aquifolium* L. was to identify the genomic affiliation of the Romanian insular population of *Ilex aquifolium* L. from the residency status point of view, as a native or as a newly introduced and cultivated species from somewhere else and acclimatized in Romania. We comparatively analyzed the characteristics of the whole-genome (SNPs) between the RO population with two recognized resident native populations (BG and SR) and with one cultured population (HU). As such, we set out to identify if the RO population is oriented toward a native population or a cultivated one from the point of view of its genomic structure. It should also be mentioned that these three locations selected for the *Ilex aquifolium* L. sample collection (HU, SR, and BG) are the closest geographically to the Romanian insular location from the Zimbru population in the Codru-Moma Mountains, Romania.

## 2. Results

The results of the present study are divided into two categories. The first set of results were obtained by NGS sequencing and subsequent QC for the four locations from which the sampling was made. The second set of results are presented with reference to the following: (1) genomic SNP identification across all the populations of *Ilex aquifolium*, using the *Ilex asprella* **(Hook. and Arn.) Champ. ex Benth**. genome as a reference; (2) identification of positions and comparison of SNPs in genes from the four populations, at the chloroplast level, with *Ilex aquifolium* L. as a reference; (3) identification of the positions and comparison of SNPs in genes from the four populations at the level of the mitochondria, with reference to *Ilex pubescens* **Hook. and Arn**; (4) quality metrics for the primary assemblies and the alignment of genomes between the studied populations, with reference to the *Ilex asprella* **(Hook. and Arn.) Champ. ex Benth** genome.

### 2.1. Preliminary Results

#### NGS Raw Data and QC Results in All Four *Ilex aquifolium* L. Populations

The NGS data, with gross total numbers of 14,877,967,184 bases for the sample from the RO population, 14,877,794,440 bases for the HU population, 13,117,326,210 bases for SR, and 14,713,914,442 bases for the BG population, were generated. Total numbers of 98,529,584 reads for the RO population, 98,528,440 in the HU population, 86,869,710 in SR, and 97,443,142 reads for the BG population were generated.

**Figure 1:**
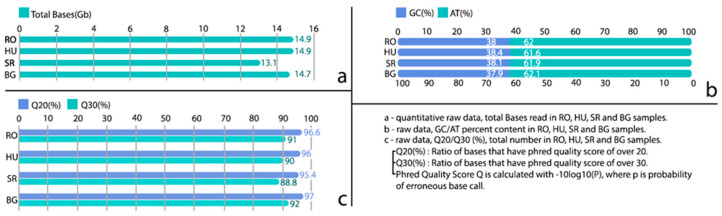
NGS raw-data statistics for all four locations (RO, HU, SR, and BG). (**a**) quantitative raw data, total bases read in RO, HU, SR and BG samples; (**b**) raw data, GC/AT percent content in RO, HU, SR and BG samples; (**c**) raw data, Q20/Q30(%) total number in RO, HU, SR and BG samples.

The GC and AT percentages had similar relative values in the four populations, with Q20 (%) and Q30 (%) base quality scores also having similar values (Figure 1).

High-quality reads were obtained at all four locations, with Q30 values between 89.4% and 92.4%. The percentages of the reads were in the range 98.4–99.0%, and well-expected values for the CG content after filtering (range: 37.8–38.3%) were obtained.

We used a modular tool to aggregate results from bioinformatics analyses (quality control, GC content, raw reads, and sequence alignments) across all the samples (RO, HU, SR, and BG). We summarized a single statistical report; more details can be found in the Supplementary Material summarized in Table S1.

### 2.2. Processed Results

#### 2.2.1. SNP Analysis

We performed the genomic SNP identification across all the populations of *Ilex aquifolium* L. (RO, HU, SR, and BG), using the *Ilex asprella* **(Hook. and Arn.) Champ. ex Benth.** genome (CUHK_Ilex_v2.1) as a reference. A similar ratio of transitions vs. transversions in SNPs was present**.** Details can be found below in Table 1.

**Figure 2:**
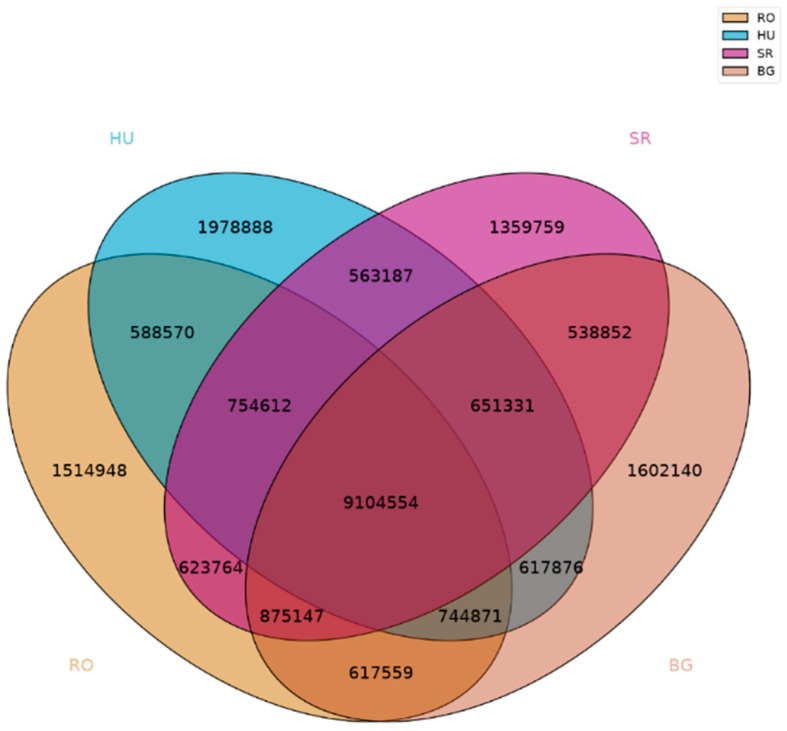
Venn diagram depiction of SNP intersections in all four datasets (RO/HU/SR/BG).

For SNP comparison in all four datasets (RO, HU, SR, and BG), a Venn diagram was generated for visualization (Figure 2). Sets of Venn diagrams were generated for paired locations, such as HU-BG, HU-SR, RO-BG, RO-HU, RO-SR, and SR-BG. For more details, see the Supplementary Material Figures S1–S6.

#### 2.2.2. Chromosome-Level Distributions of SNPs Across the Four Locations

**Figure 3:**
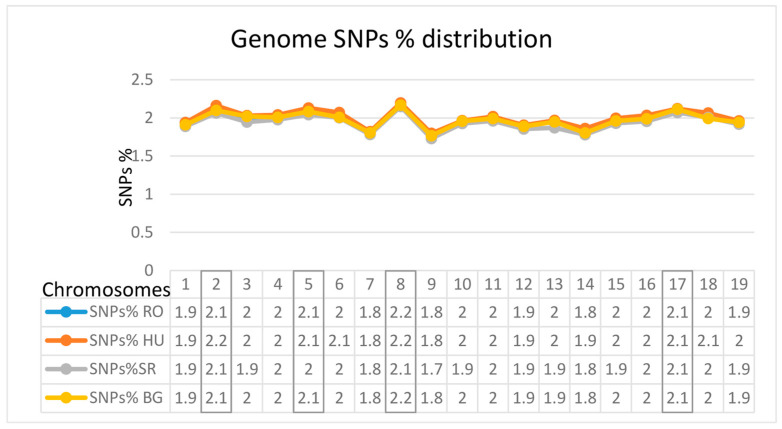
The chromosomal SNP-percentage-level representations with highest values at all four locations (RO, HU, SR, and BG).

Higher-percentage values of SNPs were obtained for chromosomes 2, 5, 8, and 17 according to the reference genome, *Ilex asprella* **(Hook. and Arn.) Champ. and Benth.** genome (CUHK_Ilex_v2.1) (Figure 3).

#### 2.2.3. Identification of SNP Positions in Genes at the Four Locations at the Levels of Chloroplasts and Mitochondria

According to our analysis at all four locations, we identified a cumulative total of 14 chloroplast genes that presented SNPs. Of these, six genes (*psbA*, *rpoB*, *clpP*, *rpl14*, *rpl16*, and *ndhD*) were present at all four locations. Three of these genes were highlighted with a higher number of SNPs: *clpP*, *rpl14*, and *rpl16*. Also, a differentiation into two categories of SNPs as the number per gene identified was observed, with similar values for samples from spontaneous populations compared to the cultivated population (Table 2).

At all four locations, we identified a higher cumulative number of genes with SNPs in mitochondria than in chloroplasts, namely, in a total of 17 genes. Of these, 12 genes (*rrn18*, *nad4*, *atp4*, *nad2*, *cob*, *nad7*, *atp1*, *nad1*, *nad1*, *matR*, *rrn26*, and *atp9*) were present at all four locations. Of these, the nad2 and rrn26 genes presented higher numbers of SNPs. An equal number of SNPs were identified in seven genes. Here, too, the differentiation of SNP values into two categories is observed, correlated with the population status in terms of the origin: native or cultivated (Table 3).

#### 2.2.4. Quality Metrics for the Primary Assemblies

The representative statistical data generated with reference to the genomic alignments among the four locations in the HTML report are presented in Table 4.

#### 2.2.5. Modeling and Graphical Representation of Genomic Alignments

**Figure 4:**
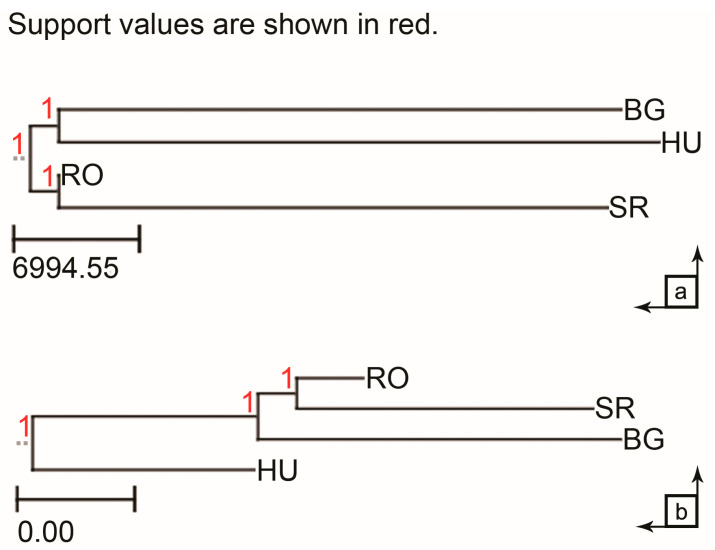
Phylogenetic trees based on (**a**) breakpoint distance; (**b**) substitution distance—for all four samples.

According to the genomic analysis between the individuals at the four locations, we generated phylogenetic trees that showed the existing interrelationships between them. According to the analysis, a common origin is identified for the RO-SR clade (in the case of the breakpoint distance) and a clade with a common ancestor among the three spontaneous populations (in the case of the substitution distance). Within this clade, there is a sub-clade with a common ancestor between RO and SR. In both genomic analyses, the common ancestor between the populations from RO and SR is identified, as shown in Figure 4.

## 3. Discussion

The events with importance in evolution and that lead to the emergence of genetic diversity are mutations, genetic drift, gene flow, and natural selection, an important role being played by the interaction between the environment and genotypes, with reference to genetic diversity [41,42]. The quantification of these events with importance in evolution (implicitly in genetic diversity) can be performed by analyzing the allelic variation, the proportion of polymorphic loci, the average allelic value/locus, the polymorphism/rate/frequency of the polymorphism at the SNP level, etc.

### 3.1. SNP Population Structure

In the present study, the analysis of the SNPs at the genome level in samples of *Ilex aquifolium* L. identified the existing differences among the local populations in RO, SR, and BG (from spontaneous flora) and HU (cultivated). The HU (cultivated) population has a higher number of SNPs (with approximately half-a-million SNPs/genome) compared to those in the other three populations (RO, SR, and BG). Similar intra-population studies have shown that plant-genome-based SNP numbers, in compared populations (cultivated vs. spontaneous), have higher levels of polymorphism in cultivated populations compared to those in native ones [43].

Venn diagrams showing the SNPs found among the four locations reveal the fact that the RO location of *Ilex aquifolium* L. has the highest number of SNPs in common with the SR location (11,358,077) and decreasing numbers in common with the BG (11,342,131) and HU (11,192,607) locations. Given the highest level of the SNP-based similarity between RO and SR, we believe that the location in RO, with an extremely compact, non-dispersed location, has a connection or a common ancestor with the population of SR. The population of RO, because of the extremely compact area (Supplementary Materials Figure S7), does not show individuals scattered in the forest area, as would be expected. Also, the RO population is found in only one very limited location, which leads us to hypothesize the existence of a possible anthropic factor responsible for the presence of the *Ilex aquifolium* L. species at this location in RO. From the point of view of the origin of the insular area in the RO population, we propose that it is likely to have a common ancestor with the local population from SR according to the number of SNPs found in common during the SNP analysis at the genome level.

### 3.2. The Distributions of SNPs at the Chromosomal Level

Based on the analyses that we carried out, the largest genomic differences were found on chromosomes 2, 5, 8, and 17, as shown in Figure 3. Sequencing studies and chromosomal analysis in *Arabidopsis thaliana* **(L.) Heynh.** [44], on chromosome 2, have shown that the genes/proteins expressed by this chromosome have regulatory functions of signal transduction, of transport, and related to the binding of proteins. Similarly, for chromosome 5 [45], the proteins expressed play roles in metabolism (21.1%), transcription (18.6%), and defense (11.9%). On chromosome 8 in rice [46], genetic regions (between RM6999 and RM22529) are involved in the rate of photosynthesis and are important in the hydraulic conductance of plants, root length, root function, surface area, and stomatal conductance. SNPs with locations on chromosome 17 may be important in relation to the *HaSPL* genes, which play crucial roles in the flowering process, stem/root tissue development, as well as in the plant’s responses to abiotic stress factors [47].

### 3.3. Identification of SNP Positions on Genes at the Four Locations at the Level of the Chloroplast and Mitochondrion

#### 3.3.1. Chloroplastidial SNPs

Higher SNP counts were identified at the level of some chloroplastidial genes, *rpl14/16*—chloroplast genes encoding a ribosomal protein (L14/16), which are constituents of the large ribosomal subunit. High SNP numbers in these genes may indicate genetic variation affecting ribosomal function, which could impact plant growth, development, or stress responses. Mutations in *clpP*—the proteolytic subunit of the ATP-dependent Clp protease—may lead to altered protein turnover, degradation, or repair. Disruptions in ClpP function could impact chloroplast homeostasis, photosynthesis, and overall plant health. Regarding comparative values between the spontaneous flora locations (RO, SR, and BG) and the cultivated location (HU), the presence of SNPs was observed in the genes *psbK* (Photosystem II reaction center protein K), *rpoC1* (β-subunit of RNA polymerase), *ccsA* (involved in cytochrome c biogenesis within the chloroplast), *ndhA* (NADH dehydrogenase subunit A), and *ycf1* (translocon at the inner envelope membrane of chloroplasts 214 and *ycf1.2*) and the absence of SNPs in the cultivated population. High SNP values for *psbK* might affect PSII function, electron transport, or stability; an altered *rpoC1* function could affect the overall plant development and stress tolerance. For *ccsA*, high SNP values might disrupt cytochrome c assembly or function; for *ndhA*, SNPs in this gene may affect electron transport and energy conversion. Altered *ndhA* function could impact plant growth and stress responses. High SNP values for *ycf1* might influence plastid biogenesis, protein translation, or RNA processing. Implications could range from altered chloroplast structure to overall plant fitness. Based on chloroplast gene mutations, changes are observed that intervene in energy metabolism correlated with genes involved in plant growth and responses to abiotic stresses.

#### 3.3.2. Mitochondrial SNPs

From the analysis of the seventeen mitochondrial genes, three main directions were identified.

In the first instance (with *Ilex pubescens* **Hook. and Arn** as a reference) two clusters are individualized among all four populations, with relatively uniform SNP values among the native flora (RO, SR, and BG) locations compared to the cultivated location (HU).

In the second case, similar values for the RO-SR-BG cluster were evident for genes such as *rrn18* (mitochondrial 18S ribosomal RNA, a component of the 30S small subunit of mitochondrial ribosomes), *cox2* (cytochrome c oxidase subunit 2), *rps3* (nuclear ribosomal protein S3, implicated in the assembly of the ribosomal small subunit), *nad2* (NADH dehydrogenase subunit 2), *atp9* (subunit 9 of mitochondrial F0-ATPase), *nad7* (NADH dehydrogenase subunit 7), *rps1* (nuclear ribosomal protein S1), *nad1* (NADH dehydrogenase subunit 1), and *rrn26* (mitochondrial 26S ribosomal RNA protein).

In the third case, the genes can be clustered into a group in relation to ribosomal function and protein biosynthesis, as well as a second group that contains genes with importance in energy metabolism/electron transport chain–ATP synthase.

Thus, in the ribosomal function group, the following genes are identified: SNPs in *rrn18/26* can impact ribosomal function, which, in turn, affects protein synthesis within the cell; SNPs in *rps3* can influence protein synthesis and cellular growth; and SNPs in *rps1* can impact protein synthesis and cellular processes.

From the electron transport chain/ATP synthase group: SNPs in *cox2* may alter the efficiency of oxidative phosphorylation; SNPs in *nad2/7/1* may impact energy metabolism; and SNPs in *atp9* can affect cellular energy levels. Also, similar SNP values were highlighted regardless of the location. These values were identified for the genes nad4 (NADH dehydrogenase subunit 4), *atp4* (subunit 4 of mitochondrial F0F1 ATPase), *cob* (cytochrome b), *atp1* (ATP synthase subunit alpha), *nad1* (NADH dehydrogenase subunit 1), *matR* (reverse transcriptase/RNA maturase protein, involved in splicing), and *atp9* (subunit 9 of mitochondrial F0-ATPase).

The genetic variations in these genes and their SNPs contribute to plant adaptation, evolution, and responses to environmental changes, so these results could be used to study plant diversity and improve crop resilience [48,49].

At the locations of Mala-Reka SR (1033 m altitude), Borino BG (1143 m altitude), and Zimbru RO (258 m altitude), the *Ilex aquifolium* L. species is located in the forest mountain area, with low temperatures correlated with intense ultraviolet radiation, which can lead to adaptive gene changes at the mitochondrial level [50]. Saline soils and water stress affect the energy balance of plants. Mitochondrial genes may respond in order to maintain cellular homeostasis.

### 3.4. Quality Metrics of Genome Assemblies

We generated several quality metrics for the country-specific genome assemblies. These metrics are reported in Table 4 and are based on contigs of lengths longer than or equal to 500 bp. In general, the RO, HU, and BG assemblies were of similar quality, with the SR assembly slightly lagging behind. The RO assembly had the longest total length, at 604 Mbp distributed in 96,602 contigs, while SR had only 582 Mbp in total, for a total of 112,689 contigs. The longest contig was found in the RO assembly, at 147,155 bp. In terms of contiguity, the N50 metric, which represents the length (in bp) of the shortest contig at 50% of the total assembly length, had the highest value for the BG assembly (at 13,200 bp), with RO coming in at a close second, with an N50 value of 12,964 bp. Similar trends were observed for the BG and RO assemblies in terms of the N90 metric, which assesses contiguity based on 90% of the assembly. The corresponding values were 2798 for BG and 2690 for RO. The SR assembly had the lowest contiguity, with an N50 value of 10,112 and an N90 value of 2230.

### 3.5. Genomic Alignments

In this study, our goal was not to achieve a pan-genome, as was achieved, for example, in cultivated plantain species [51,52], but to understand the diversity or genomic proximity of *Ilex aquifolium* L. among the four locations. Also, we focused on the possible origin of the population in RO and its likelihood to be either a native or a cultivated variety of the species. Figure 4a shows the structural variation evolution, in the case of the four genomes, based on genomic rearrangements over time, which indicates a common origin at the genome level between the locations RO and SR and between BG and HU. According to the substitution distance phylogenetic tree from Figure 4b, the comparative analysis was followed by genomic alignment among the four locations, having as a criterion the number of SNPs existing at each location. As in the previous case (from Figure 4a), here, the results also indicate a common origin of the populations of RO and SR and these two having a common origin with the population of BG. The obvious difference is given by the fact that the cultivated population of *Ilex aquifolium* L. (HU) is on a separate branch of the phylogenetic tree compared to those at the locations with native populations.

### 3.6. Phenotypic Traits in Relation to Genomic Variability

The present study shows the genomic variability among the four locations (RO, SR, BG, and HU), indicating a possible common origin between the RO and SR locations. In our previous studies on the morphologies and phytonutrient profiles in the same populations of *Ilex aquifolium* L., it emerged that the population in HU is close to that in SR [53]. By comparing the RO and SR locations with the HU location, differences in habitat, plant habitus, and morphology can be observed. In the HU population (in a maintained park area), the location is at a lower altitude than the two locations RO and SR, which, over time, could have produced morphological adaptation changes correlated with changing climatic factors in comparison to those of the populations from RO and SR, which are located in forest areas. Even so, both genomic and morphological changes show a differentiation into two clusters, i.e., the native species populations versus the cultivated one from HU. One possible reason for this is the abiotic factors that are relatively controlled in terms of the water regime and nutrient access for individuals in the HU population.

## 4. Materials and Methods

### 4.1. Plant Harvesting

**Figure 5:**
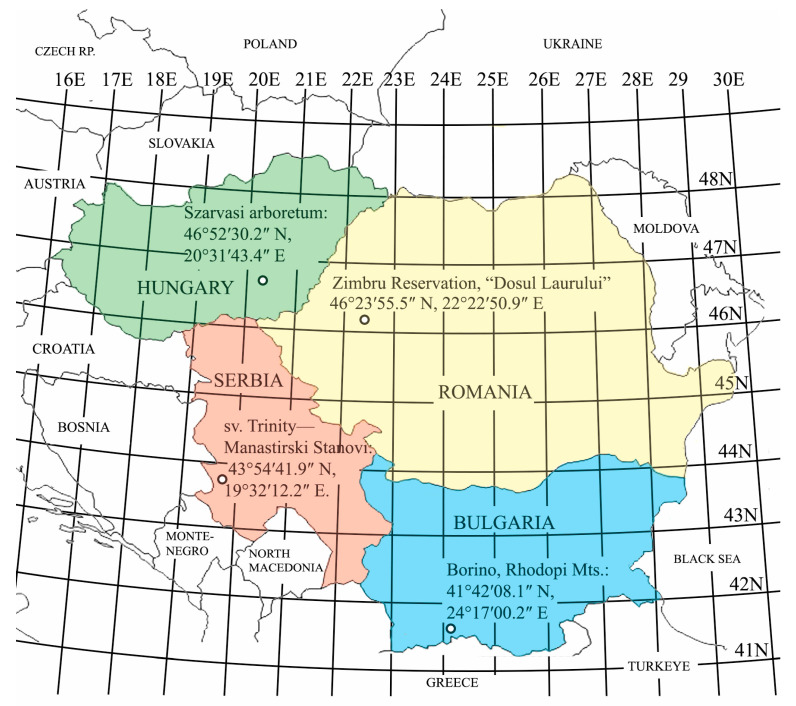
Harvesting points of *Ilex aquifolium* L. samples. Romania, Zimbru Reservation, “Dosul Laurului”, GPS coordinates of 46°23′55.5″ N, 22°22′50.9″ E; Hungary, Szarvasi Arboretum, 46°52′30.2″ N, 20°31′43.4″ E; Bulgaria, Borino, Rhodope Mountains, 41°42′08.1″ N, 24°17′00.2″ E; and Serbia, in the middle of the Mala Reka region, Sv. Trinity—Manastirski Stanovi, 43°54′41.9″ N, 19°32′12.2″ E.

All the harvested plants used in this study belong to the *Ilex aquifolium* **L**. species. The GPS coordinates of the collection points are the following: Romania, Zimbru Reservation, “Dosul Laurului”, GPS coordinates of 46°23′55.5″ N, 22°22′50.9″ E; Hungary, Szarvasi Arboretum, 46°52′30.2″ N, 20°31′43.4″ E; Bulgaria, Borino, Rhodope Mountains, 41°42′08.1″ N, 24°17′00.2″ E; and Serbia, in the middle of the Mala Reka region, Sv. Trinity—Manastirski Stanovi, 43°54′41.9″ N, 19°32′12.2″ E, as shown in Figure 5. At each collection point, 5 shoots, with the approximate age of 1 year, were harvested from female plants, in the 11 AM–2 PM interval during November 2022. The collected samples were wrapped in aluminum foil to avoid contamination and kept at a temperature of −80 °C before their processing.

### 4.2. DNA Extraction and Sequencing

The genomic DNA (gDNA) of *Ilex aquifolium* L. was extracted from an amount of 50 mg of fresh leaves preserved on dry ice (4 extractions, separated for each location) followed by WGS for all four locations.

Genomic DNA extractions for all the samples were performed using DNeasy plant mini purification kit from Qiagen (S.C. Omnivet Impex SRL/OmniGen, Bucharest, Romania). The DNA quality control (QC) method—DNA quantification was performed with Quant-iT™ PicoGreen™ dsDNA assay kits (Thermo Fisher Scientific Inc., BERD Trading Ltd., Bucharest, Romania) and dsDNA reagents from Invitrogen™, using Victor 3 fluorometry (Perkin-Elmer, Lisboa, Portugal). The DNA condition assessment was performed using the gel electrophoresis method. The DNA size check for DNA fragments <1 kb was performed with a 2100 Bioanalyzer and a DNA 1000–7500 chip for normal PCR products.

For NGS analysis, a Truseq PCR-free (350 bp short reads) library preparation kit was used. An Illumina system (Sequencer/Novaseq 6000), a read length of 151, a paired-end (2 × 150 bp) read type, and 12 Gb/sample (at each location) were used. The sequencing was performed by Macrogen Ltd., Seoul, Republic of Korea. 

Library QC Method—The size of the PCR-enriched fragments was measured by checking the template size distribution on an Agilent Technologies 2100 Bioanalyzer using a DNA 1000 chip. The library quantity check was performed using an Illumina library.

Considering the size of the *Ilex asprella* **(Hook. and Arn.) Champ. ex Benth.** genome is 835 Mb, the sequencing read depth coverage was estimated to be at least 12×. The raw-genomic-archived FastQ sequences resulted in NGS for all four locations and are indexed in the NCBI SRA under BioProject accession number PRJNA941469.

The experiments of the gDNA extractions and NGS services for WGS—short reads—were performed by Macrogen Europe and its affiliate Macrogen Seoul, Republic of Korea. The sample shipment was made on dry ice.

### 4.3. WGS Analysis

The raw NGS data files, RO_1.fastq.gz, RO_2.fastq.gz, HU_1.fastq.gz, HU_2.fastq.gz, SR_1.fastq.gz, SR_2.fastq.gz, BG_1.fastq.gz, and BG_2.fastq.gz, were deposited in the NCBI SRA database under BioProject accession number PRJNA941469.

The raw sequenced reads were checked with FastQC v. 0.11.9 [54], and all the quality control tests were passed by the four samples. Next, decontamination was performed using Kraken2 v. 2.1.2 [55] on a prebuilt database available online, denoted as “PlusPF”, and built on 14.03.2023 from RefSeq sequences of archaea, bacteria, viruses, plasmids, humans, protists, and fungi [56]. Decontamination was then performed using the following settings: “--memory-mapping --paired --gzip-compressed --use-names --minimum-hit-groups 4 --confidence 0.1”. Classified reads were discarded, and unclassified reads were retained and used for all the further analyses.

MaSuRCA (Maryland Super Read Cabog Assembler) v. 4.1.0 [57] was used to assemble the decontaminated reads into contigs for each of the 4 samples. QUAST v. 5.2.0 [58] was then used to compute various quality metrics for the primary assemblies and RepeatModeler and were used to identify and mask repetitive sequences, respectively. The latter two software packages were obtained via the TETools Docker image, v. dfam/tetools 1.8 [59]. For the first joint variant-calling analysis, we used the Ilex Asprella assembly, version CUHK_Ilex_v2.1 (NCBI ID GCA_023539305.1), as a reference. The Sarek pipeline, v. 3.1.2, from nf-core [60] was used to identify small variants (SNPs and indels) for each of the four samples relative to the Ilex *asprella* reference. Some of the main steps implemented in the pipeline relied on the following software packages: BWA v. 0.7.17-r1188 [61], for mapping reads to the reference via the bwa mem command; GATK v. 4.3.0.0 [62], for marking duplicates and identifying variants via the MarkDuplicates and HaplotypeCaller subroutines, respectively; Samtools v. 1.16.1 and Bcftools v. 1.16 [63], for quality control and various maintenance operations on mapped reads and identified variants, respectively; and MultiQC v. 1.13 [64], for integrating various statistics and results from the different pipeline stages. The Nextflow framework, v. 23.04.0 [65], was responsible for running the pipeline. The result was a consolidated VCF file, with variants called in the four NGS samples.

For the chromosome-level distributions of SNPs across the four locations—*Ilex asprella* chromosomes (based on the *Ilex asprella* **(Hook. and Arn.) Champ. ex Benth.** genome CUHK_Ilex_v2.1 as a reference)—the SNP counts for each individual chromosome were computed using basic Unix tools, such as grep, sort, uniq, and sed. In the next step, the percentages of SNPs obtained for all 19 chromosomes at the four locations were calculated as the ratio between the length of the chromosomes (expressed in nucleotides) and the number of SNPs obtained in the previous analysis, see the Supplementary Material (Table S2).

For the second joint variant-calling analysis, we used the *Ilex aquifolium* L. chloroplast assembly (NCBI ID NC_068798.1) as a reference, based on the same pipeline as previously described. After obtaining the VCF file, specific variants were retained, which overlapped the 86 annotated genes (as of 01.02.2023) for this chloroplast sequence [66].

Finally, for the third joint variant-calling analysis, we acquired the *Ilex pubescens* **Hook. & Arn**. mitochondrion sequence from NCBI (accession NC_045078.1) and used it as a reference. As in the chloroplast analysis, we first selected all the biallelic SNPs, after which, for a separate analysis, we retained only those that overlapped the 54 annotated genes (as of 01.02.2023) found in NCBI [67].

In order to generate Venn and UpSet plots, biallelic SNPs were extracted using Bcftools for the following three scenarios:

- SNPs of RO, HU, SR, and BG, relative to the *Ilex asprella* **(Hook. & Arn.) Champ. ex Benth** genome;
- SNPs of the four samples, relative to the *Ilex aquifolium* L. chloroplast sequence;
- From the previous scenario, only those SNPs that overlapped the 86 annotated genes.

Venn and UpSet plots were generated using Intervene v. 0.6.5 [68].

The overlaps between the identified SNPs and the genes from the *Ilex pubescens* **Hook. & Arn.** mitochondrion (NCBI accession NC_045078.1 used as a reference) and the *Ilex aquifolium* L. chloroplast (NCBI accession NC_068798.1 used as a reference) were computed using the “bedtools intersect” command for each location individually.

The genome alignment was carried out using the *Ilex asprella* **(Hook. & Arn.) Champ. ex Benth.** genome (CUHK_Ilex_v2.1) as a contig-naming reference.

The Minigraph–Cactus pipeline [69] implemented in the Cactus software package, v. 2.5.1, was used to create a multiple genome alignment of the 4 primary assemblies with masked repeats. The resulting HAL assembly was then used to infer 2 phylogenetic trees: the first one was created using Ragout v. 2.3 [70] and based on breakpoint distances (e.g., from inversions, translocations, or other chromosomal rearrangements, as depicted in Figure 4a), while the second tree was based on substitution distances (i.e., numbers of SNPs, as shown in Figure 4b) and inferred via the phyloFit method from the PHAST package [71]. The value “1,000,100” was used for the synteny block size in Ragout, while the phylogenetic tree structure “((BG, HU),(RO,SR));” was used for PHAST. The latter choice was based on the results of the Venn diagrams from the nf-core/Sarek analyses, as outlined in the Results section.

Unless otherwise specified, all the tools that were mentioned in this section were used with their respective default settings.

**Figure 6:**
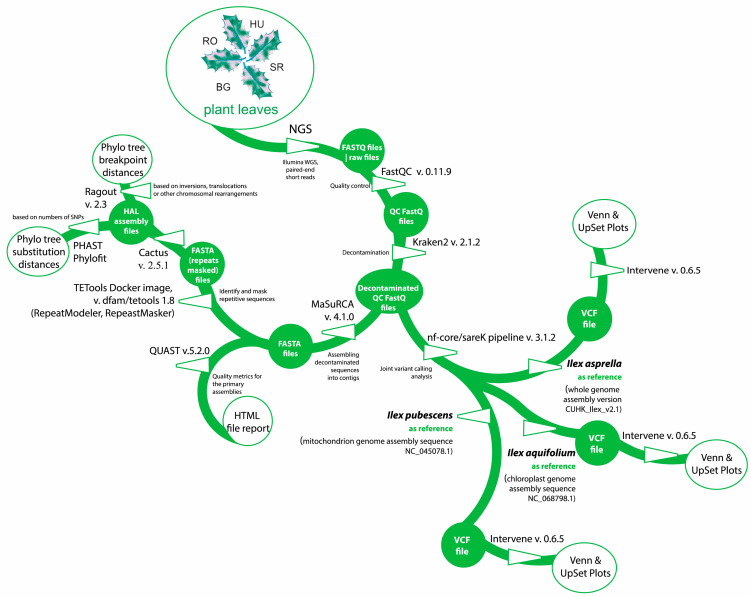
Depiction of NGS data analysis pipeline for *Ilex aquifolium* L. at all four locations.

An overview of the pipeline supported by the current analysis, including all the programs and plant genomes used as references, can be seen below in Figure 6.

## 5. Conclusions

The study of genomic variability through comparative analysis at the genome level within the four populations (three native populations (RO, SR, and BG) and one cultivated population (HU)) identified correlations based on whether they were phylogenetically close or distant. The highest number of SNPs in common was found between the RO and SR locations. SNPs identified in all four populations showed higher frequences for 4 chromosomes (2, 5, 8, and 17), this being in correlation with cellular regulatory functions, transport of metabolites/water, and responses of the plants to abiotic stress factors. Similar observations for SNPs’ positions on chloroplast and mitochondrial genes were found in all four populations. Finally, according to the genome alignments, the common origin of the SR-RO populations is reconfirmed.

In accordance with the data presented in this study, at the genome level within the populations of SR, RO, BG, and HU of the species *Ilex aquifolium* L., we propose that the origin of the population in RO is common with that of the population in SR and different from that of the population of the cultivated variant in HU.

## Figures and Tables

**Table 1:** Transition (Ts) vs. transversion (Tv) ratios in SNPs at all four locations.

Sample	Vars	SNPs	Indels	Ts/Tv
BG	14,752,330	14,752,330	0	1.72
HU	15,003,889	15,003,889	0	1.72
RO	14,824,025	14,824,025	0	1.72
SR	14,471,206	14,471,206	0	1.72

Vars, variants; Indels, insertions-deletions; RO, Romania; HU, Hungary; SR, Serbia; BG, Bulgaria.

**Table 2:** Counts of SNP positions in genes at the four locations at the chloroplastidial level.

Chr	Start	End	Gene	RO	HU	SR	BG
NC_068798.1	450	1512	*psbA*	3	4	6	5
NC_068798.1	8184	8370	*psbK*	1	0	1	1
NC_068798.1	12,575	13,831	*atpF*	1	0	1	1
NC_068798.1	21,792	24,612	*rpoC1*	3	0	2	2
NC_068798.1	24,638	27,851	*rpoB*	3	1	3	3
NC_068798.1	69,183	69,257	*trnP-UGG*	0	0	1	0
NC_068798.1	72,652	74,715	*clpP*	13	16	13	13
NC_068798.1	80,637	81,651	*rpoA*	0	0	1	0
NC_068798.1	83,439	83,808	*rpl14*	12	9	19	19
NC_068798.1	83,935	85,265	*rpl16*	20	6	28	27
NC_068798.1	117,349	118,315	*ccsA*	1	0	1	1
NC_068798.1	118,573	120,151	*ndhD*	1	1	1	1
NC_068798.1	122,601	124,838	*ndhA*	1	0	1	1
NC_068798.1	126,765	132,456	*ycf1*	1	0	2	1

Chr, chromosome; RO, Romania; HU, Hungary; SR, Serbia; BG, Bulgaria.

**Table 3:** Counts of SNP positions in genes at the four locations at the mitochondrial level.

Chr	Start	End	Gene	RO	HU	SR	BG
NC_045078.1	37,841	39,772	*rrn18*	1	3	1	1
NC_045078.1	43,064	43,138	*trnW-CCA*	0	1	1	1
NC_045078.1	56,375	58,624	*cox2*	2	0	2	2
NC_045078.1	68,915	77,567	*nad4*	3	3	3	3
NC_045078.1	158,633	159,212	*atp4*	1	1	1	1
NC_045078.1	174,865	178,373	*rps3*	0	1	0	0
NC_045078.1	216,298	221,012	*nad2*	4	2	4	4
NC_045078.1	249,304	250,486	*cob*	1	1	1	1
NC_045078.1	252,738	253,002	*atp9*	0	1	0	0
NC_045078.1	276,579	282,510	*nad7*	1	2	1	1
NC_045078.1	295,025	295,634	*rps1*	0	1	0	0
NC_045078.1	300,823	302,353	*atp1*	2	2	2	2
NC_045078.1	438,334	439,971	*nad1*	1	2	1	1
NC_045078.1	471,524	475,330	*nad1*	2	2	2	2
NC_045078.1	472,644	474,615	*matR*	1	1	1	1
NC_045078.1	483,961	487,154	*rrn26*	4	2	4	4
NC_045078.1	503,822	504,068	*atp9*	1	1	1	1

Chr, chromosome; RO, Romania; HU, Hungary; SR, Serbia; BG, Bulgaria.

**Table 4:** Genome assembly statistics at all four locations.

QUAST Stat	Location			
	RO	HU	SR	BG
All contigs	96,602	102,971	112,689	93,492
Total length (bp)	604,400,875	603,264,456	582,153,373	599,835,904
Longest contig (bp)	147,155	109,510	111,060	135,597
N50	12,964	12,102	10,112	13,200
N90	96,602	102,971	112,689	93,492

All the statistics are based on contigs of a length of at least 500 bp.

## Data Availability

Links to publicly archived datasets: BioProject Accession PRJNA941469, https://www.ncbi.nlm.nih.gov/bioproject/?term=PRJNA941469 (accessed on 6 June 2024). Links to resources: BG 1 ILLUMINA (Illumina NovaSeq 6000) run: 48.7 M spots, 14.7 G bases, 4.5 Gb downloads, Accession SRX19590379; SR 1 ILLUMINA (Illumina NovaSeq 6000) run: 43.4 M spots, 13.1 G bases, 4.3 Gb downloads, Accession SRX19590378; HU 1 ILLUMINA (Illumina NovaSeq 6000) run: 49.3 M spots, 14.9 G bases, 4.7 Gb downloads, Accession SRX19590377; RO 1 ILLUMINA (Illumina NovaSeq 6000) run: 49.3 M spots, 14.9 G bases, 4.6 Gb downloads, Accession SRX19590376 (All the data links were accessed on 7 March 2023).

## Supplementary Materials

The following supporting information can be downloaded at https://www.mdpi.com/article/10.3390/ijms252413593/s1.
