# Supplementary material for: Genomic Variability Survey in Ilex aquifolium L., with Reference to Four Insular Populations from Eastern Europe
Source: Int J Mol Sci. 2024 Dec 19;25(24):13593. doi: 10.3390/ijms252413593 (PMC11677755; doi:10.3390/ijms252413593)
Supplement: Supplementary file 1 [file ijms-25-13593-s001.zip › figure caption.pdf]

Figure S1: Venn diagram for paired locations, HU-BG; Figure S2: Venn diagram for paired locations, HU-SR; Figure S3: Venn diagram for paired locations, RO-BG; Figure S4: Venn diagram for paired locations, RO-HU; Figure S5: Venn diagram for paired locations, RO-SR; Figure S6: Venn diagram for paired locations, SR-BG; Figure S7: *Ilex aquifolium* **L**. RO population, with a small compact areal.
