# Supplementary material for: Genomic Variability Survey in Ilex aquifolium L., with Reference to Four Insular Populations from Eastern Europe
Source: Int J Mol Sci. 2024 Dec 19;25(24):13593. doi: 10.3390/ijms252413593 (PMC11677755; doi:10.3390/ijms252413593)
Supplement: Supplementary file 1 [file ijms-25-13593-s001.zip › Table S1 QC data results.pdf]

Table S1. QC data results.

Sample Name	Percentage of reads > Q30 after filtering	GC content after filtering	Percent reads passing filters	% Mapped Reads
**BG**	92.4%	37.8%	99.0%	93.5%
**RO**	91.4%	38.0%	98.9%	93.6%
**SR**	89.4%	38.0%	98.4%	92.7%
**HU**	90.5%	38.3%	98.5%	90.5%

RO, Romania; HU, Hungary; SR, Serbia; BG, Bulgaria
