# Supplementary material for: Genomic Variability Survey in Ilex aquifolium L., with Reference to Four Insular Populations from Eastern Europe
Source: Int J Mol Sci. 2024 Dec 19;25(24):13593. doi: 10.3390/ijms252413593 (PMC11677755; doi:10.3390/ijms252413593)
Supplement: Supplementary file 1 [file ijms-25-13593-s001.zip › Table S2 Percentage distribution of SNPs at the chromosome level in the entire genome in Ilex aquifolium between the four locations.pdf]

**Table S2.** Percentage distribution of SNPs at the chromosome level in the entire genome in *Ilex aquifolium* between the four locations.

| CHR name NCBI | CHR No. | SNPs% RO | SNPs% HU | SNPs% SR | SNPs% BG |
|---|---|---|---|---|---|
| CM042184.1 | 1 | 1.938057 | 1.939134 | 1.890698 | 1.911305 |
| CM042185.1 | 2 | 2.107934 | 2.16225 | 2.065989 | 2.099675 |
| CM042186.1 | 3 | 2.008898 | 2.029526 | 1.946579 | 2.019484 |
| CM042187.1 | 4 | 2.015183 | 2.038815 | 1.975596 | 2.006924 |
| CM042188.1 | 5 | 2.088732 | 2.131365 | 2.043494 | 2.083595 |
| CM042189.1 | 6 | 2.043495 | 2.07003 | 2.001396 | 2.007639 |
| CM042190.1 | 7 | 1.812568 | 1.82099 | 1.781414 | 1.805797 |
| CM042191.1 | 8 | 2.170377 | 2.197266 | 2.149579 | 2.167811 |
| CM042192.1 | 9 | 1.773302 | 1.796861 | 1.730793 | 1.767305 |
| CM042193.1 | 10 | 1.950117 | 1.965662 | 1.925573 | 1.957698 |
| CM042194.1 | 11 | 2.001847 | 2.017094 | 1.96118 | 1.991368 |
| CM042195.1 | 12 | 1.886057 | 1.905737 | 1.856181 | 1.889694 |
| CM042196.1 | 13 | 1.9539 | 1.968227 | 1.871191 | 1.948185 |
| CM042197.1 | 14 | 1.824781 | 1.861116 | 1.778439 | 1.803134 |
| CM042198.1 | 15 | 1.970613 | 1.997165 | 1.930889 | 1.961294 |
| CM042199.1 | 16 | 2.00124 | 2.032857 | 1.955469 | 1.989292 |
| CM042200.1 | 17 | 2.119523 | 2.121979 | 2.069505 | 2.115464 |
| CM042201.1 | 18 | 2.048436 | 2.06489 | 2.003622 | 1.992875 |
| CM042202.1 | 19 | 1.943543 | 1.962151 | 1.916237 | 1.941347 |

Chr, chromosome; RO, Romania; HU, Hungary; SR, Serbia; BG, Bulgaria
