# Supplementary figures and images for: Genomic Variability Survey in Ilex aquifolium L., with Reference to Four Insular Populations from Eastern Europe
Source: Int J Mol Sci. 2024 Dec 19;25(24):13593. doi: 10.3390/ijms252413593 (PMC11677755; doi:10.3390/ijms252413593)

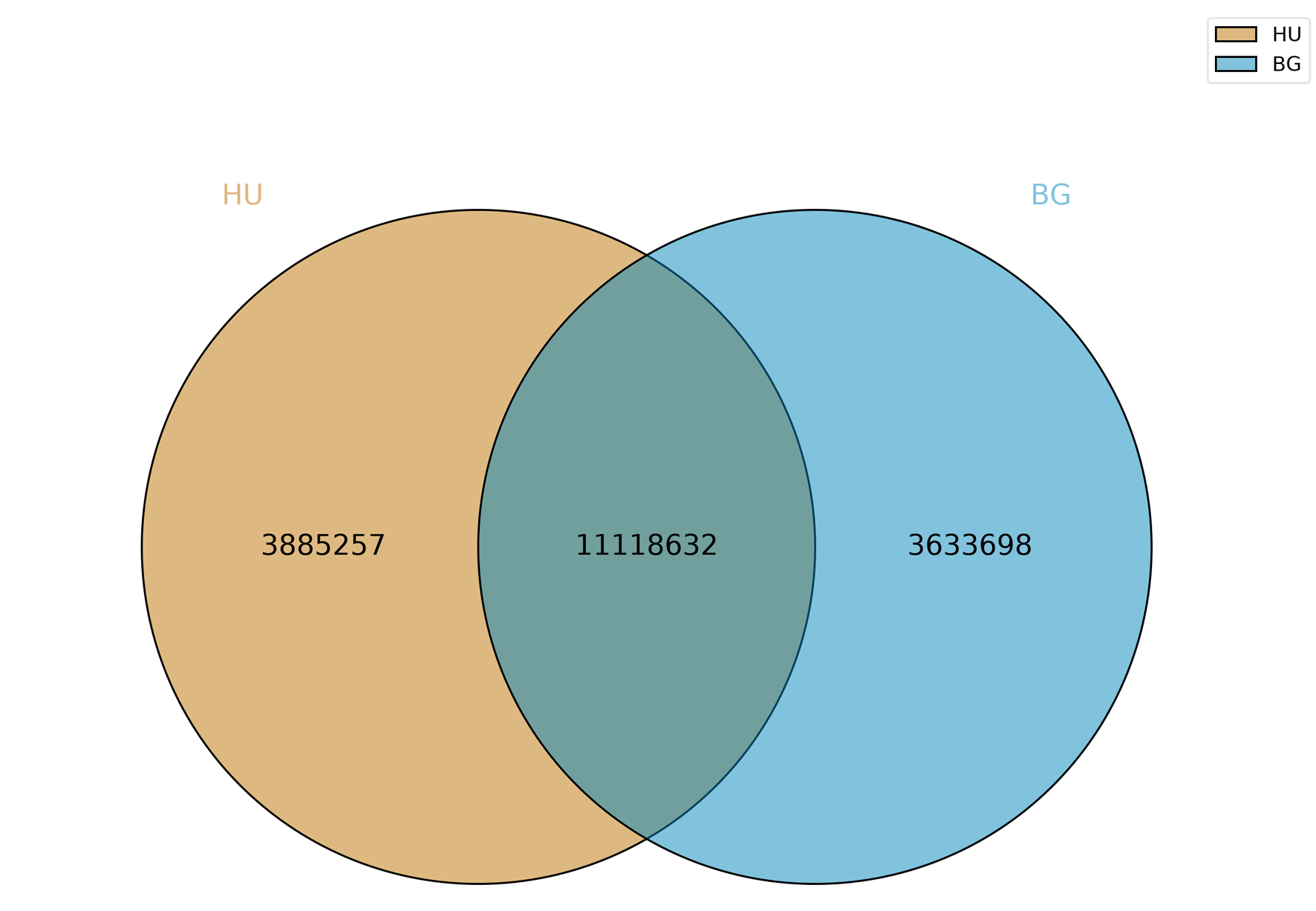

Supplement: Supplementary file 1 [file ijms-25-13593-s001.zip › Figure - S1.png]

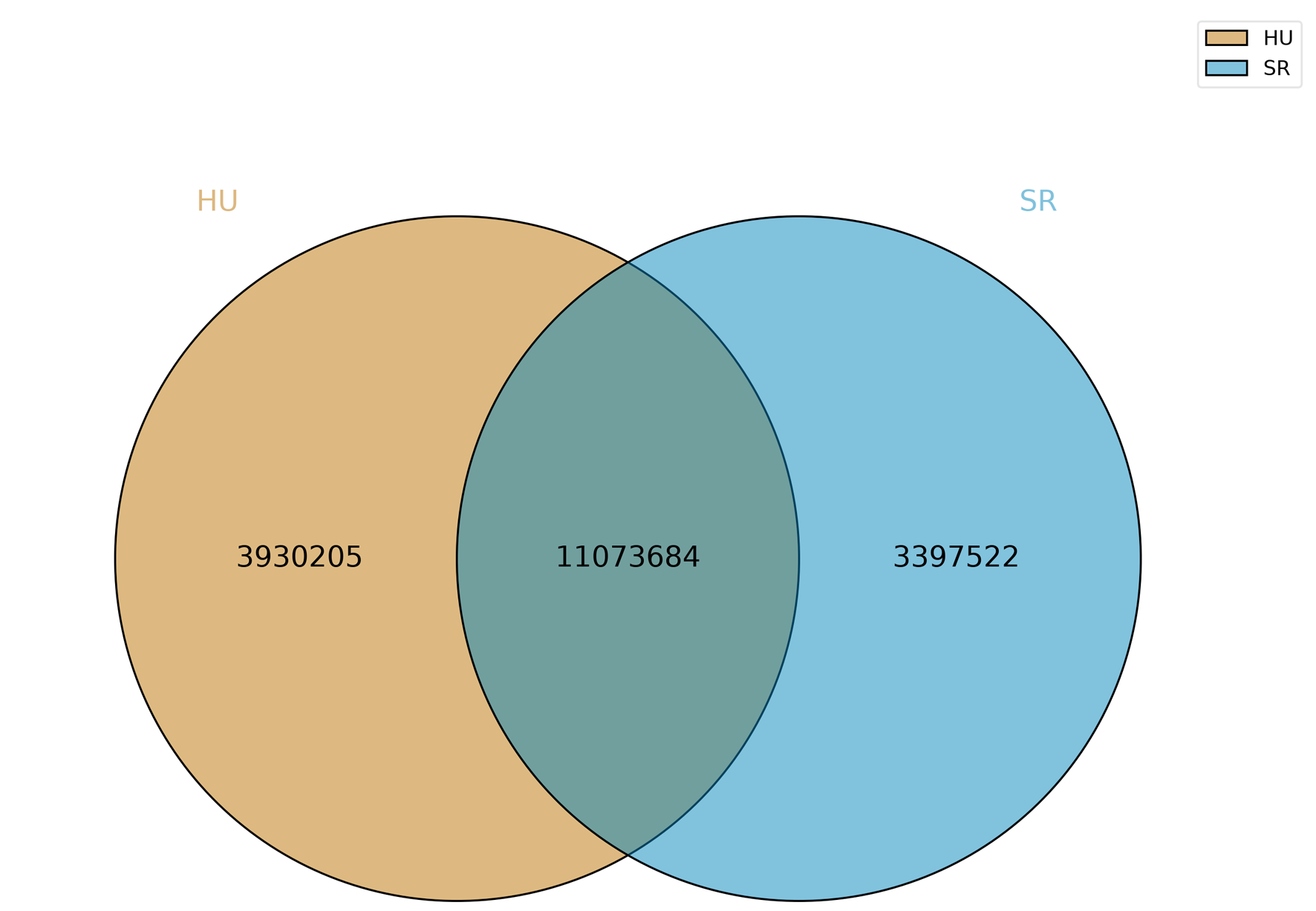

Supplement: Supplementary file 1 [file ijms-25-13593-s001.zip › Figure - S2.png]

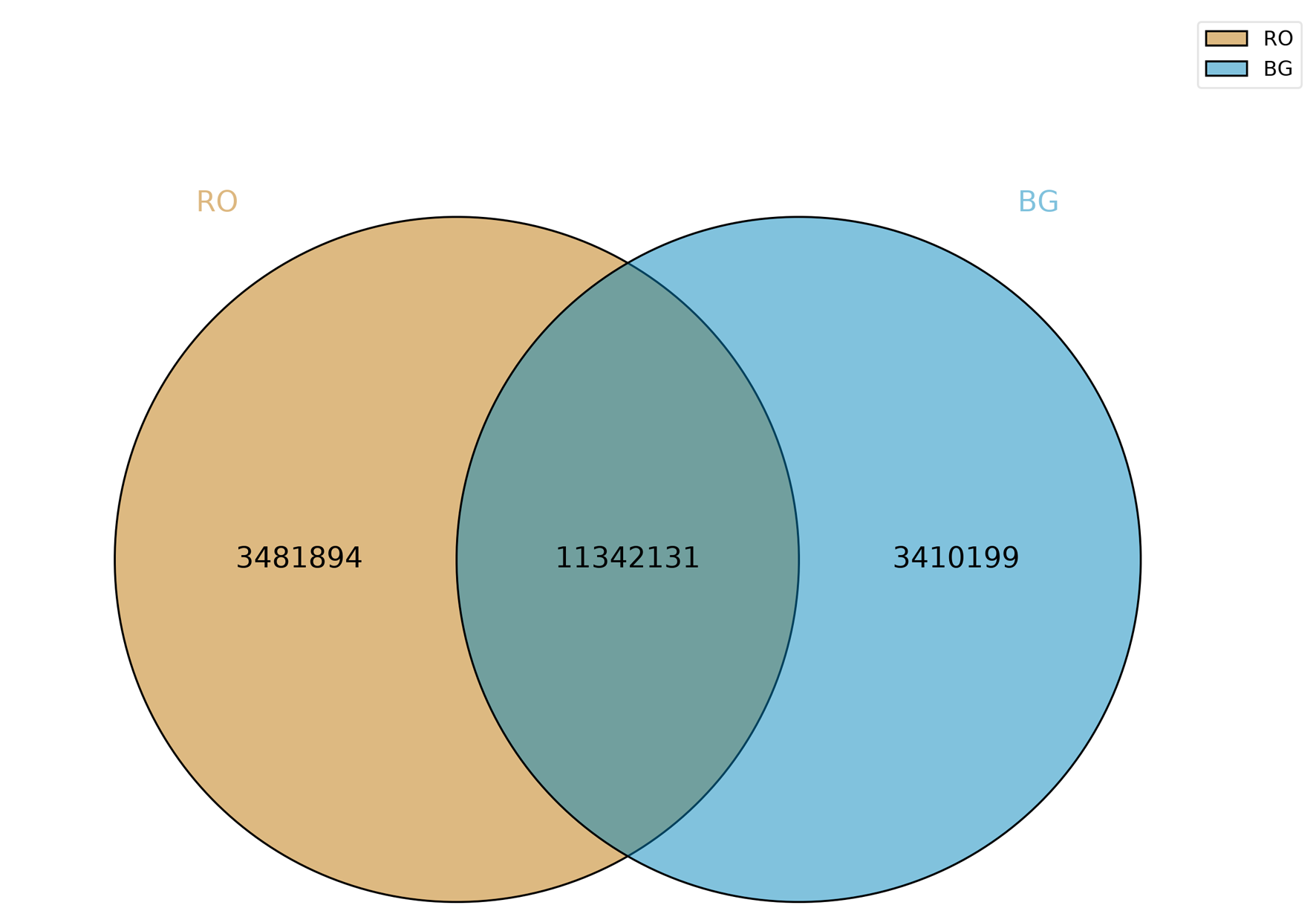

Supplement: Supplementary file 1 [file ijms-25-13593-s001.zip › Figure - S3.png]

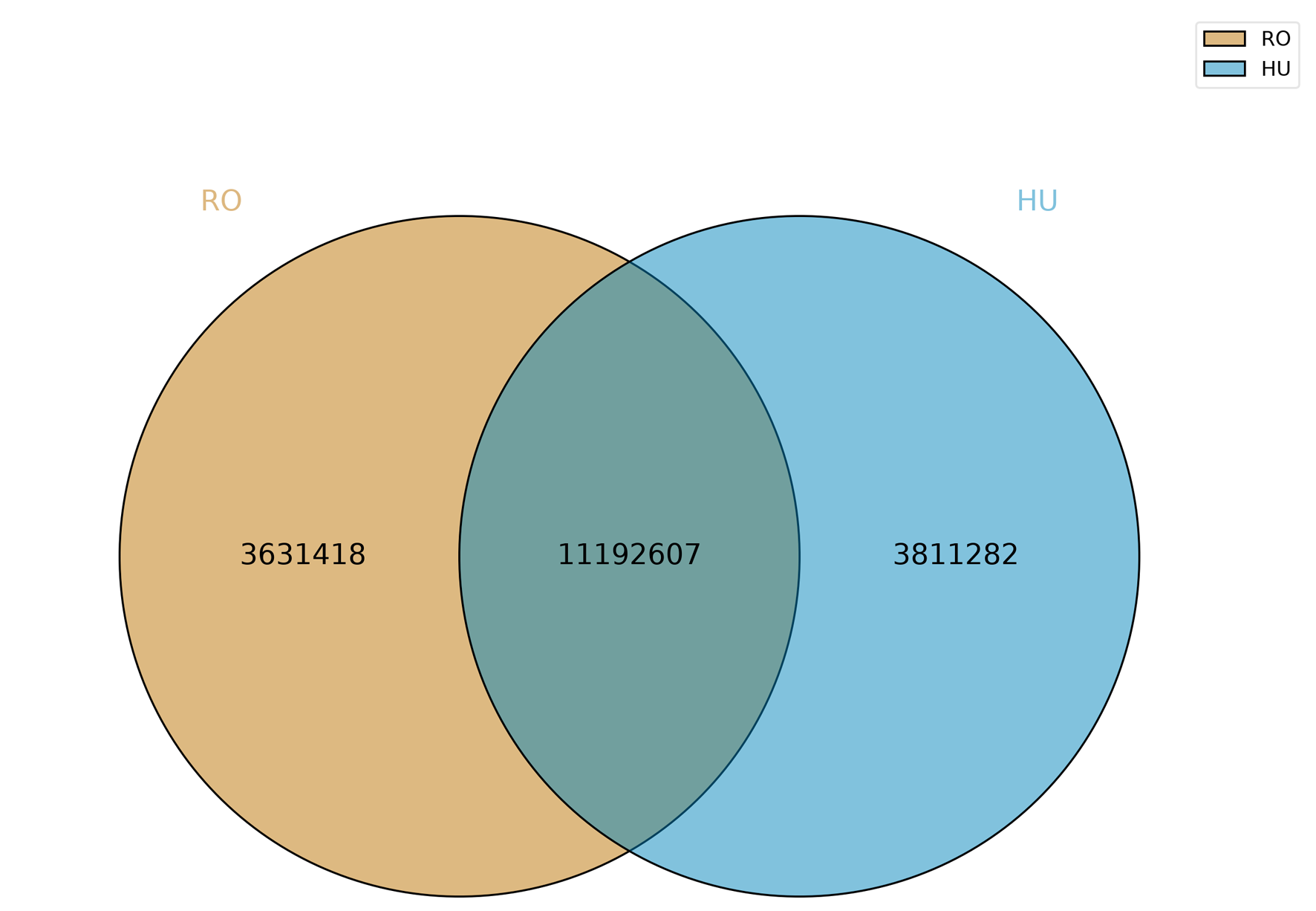

Supplement: Supplementary file 1 [file ijms-25-13593-s001.zip › Figure - S4.png]

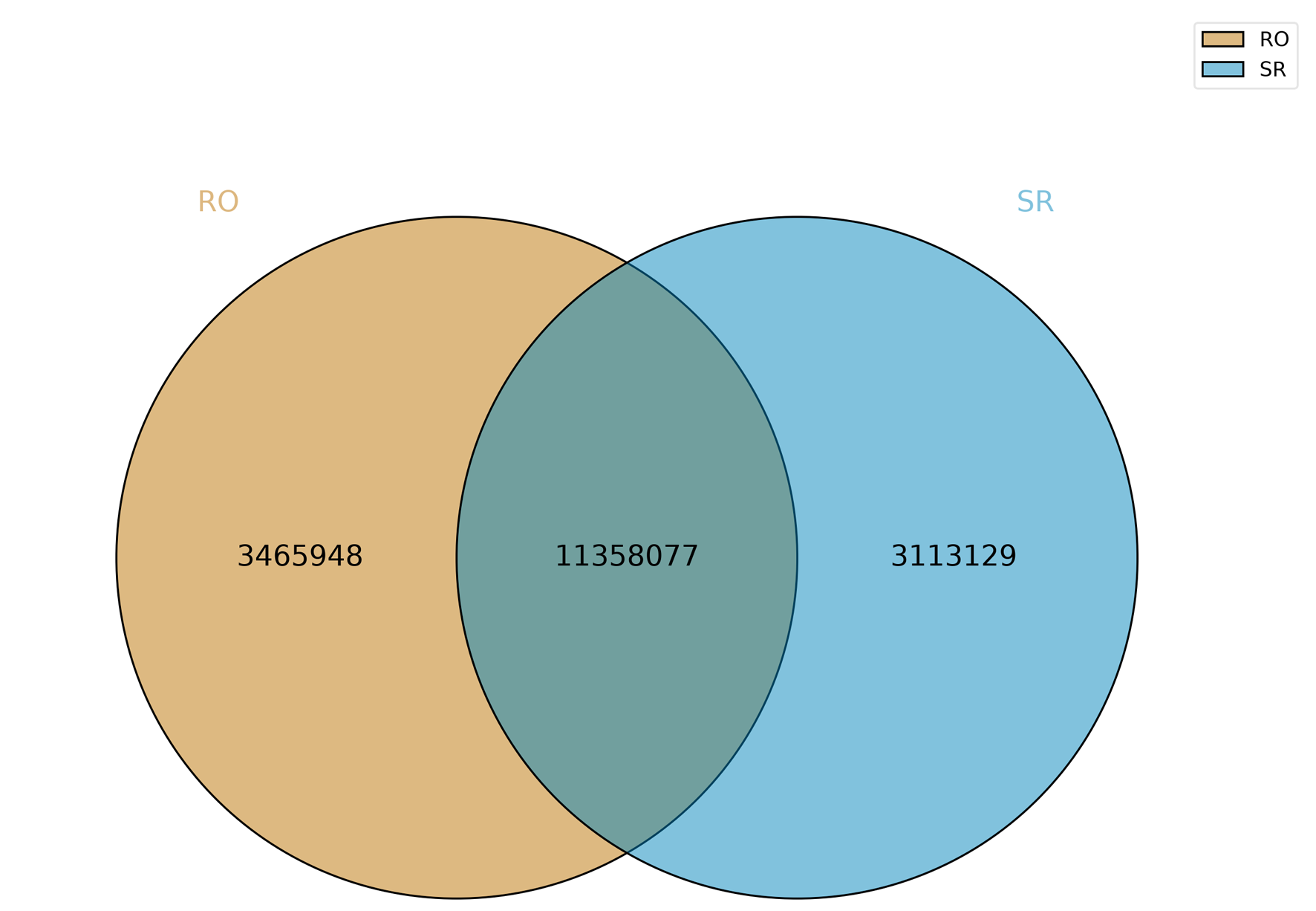

Supplement: Supplementary file 1 [file ijms-25-13593-s001.zip › Figure - S5.png]

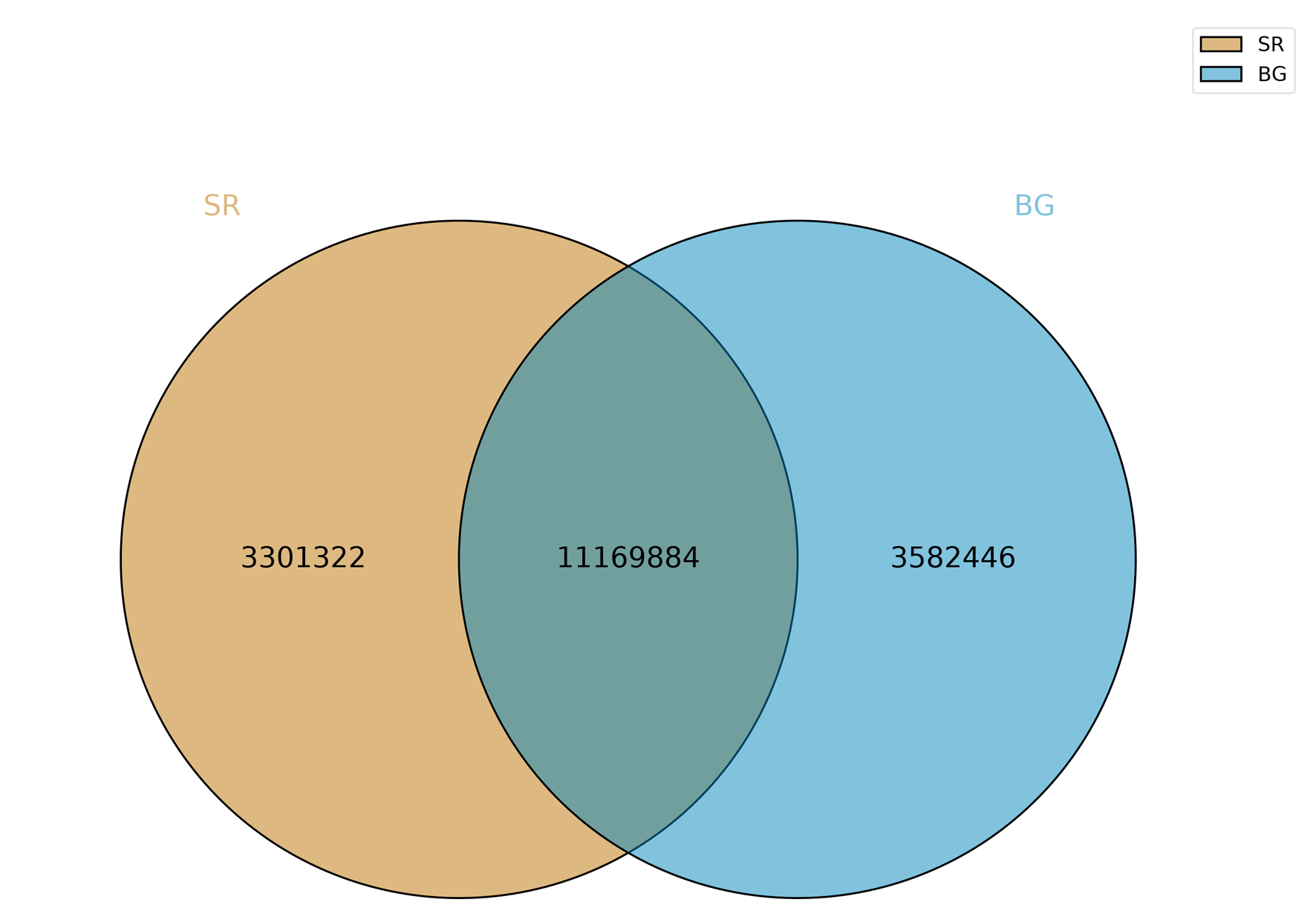

Supplement: Supplementary file 1 [file ijms-25-13593-s001.zip › Figure - S6.png]

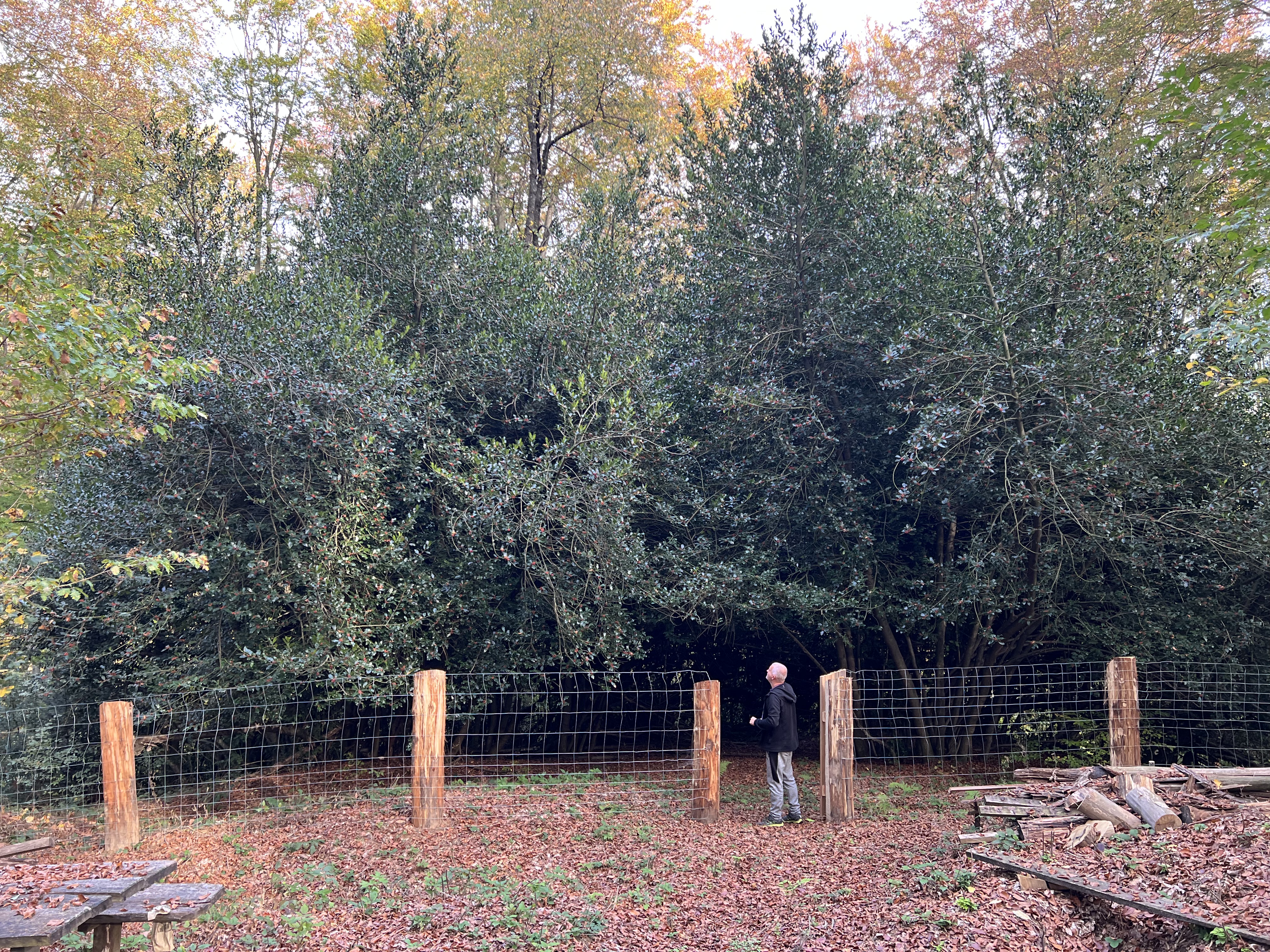

Supplement: Supplementary file 1 [file ijms-25-13593-s001.zip › Figure - S7.jpeg]
